# Performance Optimization of FA-GGBS Geopolymer Based on Response Surface Methodology

**DOI:** 10.3390/polym15081881

**Published:** 2023-04-14

**Authors:** Dazhi Wu, Junyi Wang, Tong Miao, Keyu Chen, Zilong Zhang

**Affiliations:** 1 School of Civil Engineering and Architecture, Zhejiang Sci-Tech University, Hangzhou 310018, China; 2 School of Economics, Shanghai University, Shanghai 200000, China

**Keywords:** response surface methodology, FA-GGBS geopolymer, zeolite powder, unconfined compressive strength, reaction mechanism

## Abstract

Many scholars have focused on the workability and mechanical properties of fly ash (FA)- ground granulated blast furnace slag (GGBS) geopolymer. To enhance the compressive strength of geopolymer, zeolite powder was added in the present study. A series of experiments were carried out to investigate the effect of using zeolite powder as an external admixture on the per-formance of FA-GGBS geopolymer, 17 sets of experiments were designed and tested to deter-mine the unconfined compressive strength based on the response surface methodology, and then, the optimal parameters were obtained via modeling of 3 factors (zeolite powder dosage, alkali exciter dosage, and alkali exciter modulus) and 2 levels of compressive strength (3 d and 28 d). The experimental results showed that the strength of the geopolymer was the highest when the three factors were 13.3%, 40.3%, and 1.2. Finally, a combination of scanning electron microscopy (SEM), X-ray diffraction (XRD), Fourier transform infrared spectroscopy (FTIR), and ^29^Si nuclear magnetic resonance (NMR) analysis was used to conduct micromechanical analysis and explain the reaction mechanism from a microscopic perspective. The SEM and XRD analysis revealed that the microstructure of the geopolymer was the densest when the zeolite powder was doped at 13.3%, and the strength increased accordingly. The NMR and Fourier transform infrared spectroscopy analyses revealed that the absorption peak wave number band shifted toward the lower wave number band under the optimal ratio, and the silica–oxygen bond was replaced by an aluminum–oxygen bond, which generated more aluminosilicate structures.

## 1. Introduction

The carbon emissions of the cement industry mainly come from the production process of cement clinker, namely, carbon dioxide generated by coal combustion and carbon dioxide (CO_2_) generated by the decomposition of calcium carbonate. According to data, 0.894 to 1.215 t CO_2_ will be discharged per 1 t of cement clinker produced. The CO_2_ released by cement production accounts for 7% of the total CO_2_ emission in the world [1]. In China, the production of construction materials directly or indirectly generates about 27% of the total carbon emissions. Therefore, developing low-carbon building materials is particularly important to achieve carbon peaking and neutrality. Geopolymers, as a new class of material, were first proposed by Joseph Davidovits [2]. Geopolymers are obtained through alkali excitation of inorganic materials rich in silica and aluminum components in a three-dimensional network structure, mainly inorganic SiO_4_ and AlO_4_ tetrahedra [3]. Among them, alkaline solution acts as adhesive, alkali activator, and dispersant [4]. As the raw materials of geopolymers are primarily FA, coal gangue, rice husk ash [5], and other industrial waste, compared with traditional silicate cement, they have the characteristics of high strength, high-temperature resistance, acid and alkali corrosion resistance, impermeability, and curing of metal ions. The production process is not complicated, has a low energy consumption, and is more environmentally friendly; thus, it is expected to replace the traditional silicate cement, which is characterized by a high degree of pollution and high heat release, and to become a new type of green building material [6,7].

Low-calcium fly ash (FA) is a solid waste produced by the coal combustion process, which is inexpensive, widely available, and suitable for large-scale applications. However, several experiments have shown that due to the high polymerization of glass in FA, the curing time of alkali-activated FA cementitious materials is long, and the strength of the material is low under room temperature curing [8]. Furthermore, the use of auxiliary cementitious materials as external admixtures can increase strength and improve performance [9,10,11]. Fang et al. [12] and Lee et al. [13] added ground granulated blast furnace slag (GGBS) to shorten the curing time of a FA geopolymer and significantly improved its strength. However, the addition of excessive GGBS can also lead to more considerable shrinkage and poorer crack resistance of the material. Lee et al. [13] also found that adding 10–30% GGBS can produce a denser matrix and reduce the drying shrinkage age. Therefore, the performance optimization of FA-GGBS geopolymer has become a research hotspot in the field of building materials in recent years. Developing various new high-quality cementitious materials, including natural minerals, has become a research hotspot in construction materials in recent years.

Zeolite powder is finely ground from natural zeolite rock and its color is white. Zeolite is a kind of aluminosilicate mineral with a frame-like structure formed by volcanic lava, which is mainly composed of SiO_2_, Al_2_O_3_, H_2_O, and alkali metal and alkaline earth metal ions. Among them, silica tetrahedron and alumina tetrahedron form the three-dimensional spatial frame-like structure of zeolite. Zeolite powder, a natural mineral material with high silica-alumina content, is widely distributed and has the advantages of a high specific surface area, porosity, and adsorption capacity [14]. Tay [15] uses zeolite powder as precursor. Through formal study, two ultra-lightweight geopolymer foam concrete (GFC) with density <1.5 g/cm^3^ have been successfully fabricated. Shahmansour [16] found that the compressive strength values increase in the specimens containing less than 15% zeolite powder. Ozen et al. [17] showed that zeolite is an environmentally friendly and cost-effective material suitable for making geopolymer cement. However, the studies on the synthesis of geopolymer by the alkali-activation of natural zeolites are limited and the analysis was conducted at the macro-mechanical level, and zeolite powder has not been applied to FA-GGBS geopolymer.

In order to prepare geopolymer, the preparation parameters should be determined first. However, the existing research often adopts the orthogonal test design with only simple factor analysis, which selects some representative points from the comprehensive test according to the orthogonality and cannot obtain the relationship between the various influencing factors [18]. The response surface methodology (RSM) is a statistical method that uses reasonable experimental design methods and obtains certain data through experiments, uses multiple quadratic regression equations to fit the functional relationship between factors and response values, seeks the optimal process parameters through the analysis of regression equations, and solves multivariable problems [19,20].

Consequently, in order to improve the performance of FA-GGBS geopolymer. The present research is based on response surface methodology and investigates the effect of zeolite powder as an additive on the compressive strength of FA-GGBS polymer by adding different amounts of zeolite powder. At the same time, X-ray diffraction (XRD), scanning electron microscopy (SEM), Fourier transform infrared spectroscopy (FTIR), and ^29^Si nuclear magnetic resonance (NMR) were used to study the microstructure and reaction mechanism of the composite, so as to better understand its mechanical behavior. The basic understanding of such paste obtained from the results of this study will effectively improve the compressive strength of geopolymer and reduce shrinkage.

## 2. Raw Materials

**Figure 1:**
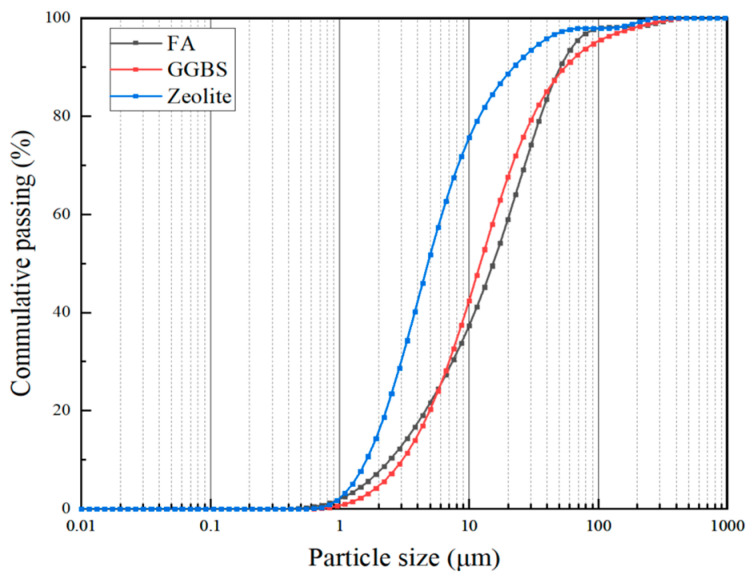
Particle size distribution of FA, GGBS, and zeolite.

The chemical compositions of the raw materials used in the tests are presented in Table 1. The fly ash (FA) used was low-calcium fly ash obtained from the Gongyi Yuanheng Water Purification Material Plant, with a specific surface area of 368 m^2^/kg. The ground granulated blast furnace slag (GGBS) was obtained from Henan Yuanheng Environmental Protection Engineering Co., Ltd., with a specific surface area of 412 m^2^/kg. The zeolite powder was obtained from the Gongyi Hengxin Filter Media Plant with a specific surface area of 698 m^2^/kg. The particle size distribution of FA, GGBS and zeolite powder is shown in Figure 1. Their average diameter is between 5–10 microns and the particle size of zeolite powder is smaller than that of FA and GGBS, which also confirms that the specific surface area of zeolite powder is much higher than that of FA and GGBS. The alkali exciter was water glass Na_2_SiO_3_nH_2_O, and the water glass modulus (as shown in Table 2) was adjusted by adding pure solid NaOH.

## 3. Experimental Design

**Figure 2:**
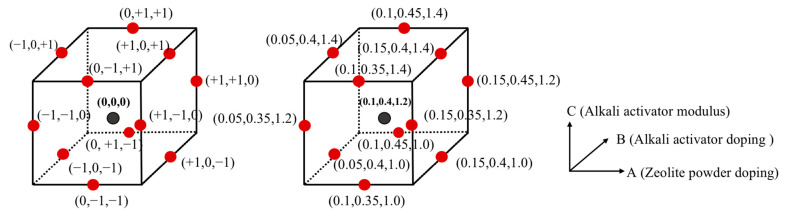
Experimental analysis model of Box–Behnken.

A series of experiments were conducted using the Box–Behnken design method for response surface methodology. The experimental analysis model is shown in Figure 2 [21]. For the sake of elaboration, the zeolite powder dosage, alkali exciter dosage, and alkali exciter modulus are denoted by the letters A, B, and C, respectively. Based on preliminary tests, the selected zeolite powder dosage, alkali exciter dosage, and alkali exciter modulus were 5–15%, 35–45%, and 1.0–1.4, respectively, and the low-, medium-, and high-level coding values are indicated by –1, 0, and 1, respectively.

## 4. Specimen Preparation and Test Methods

### 4.1. Specimen Preparation

**Figure 3:**
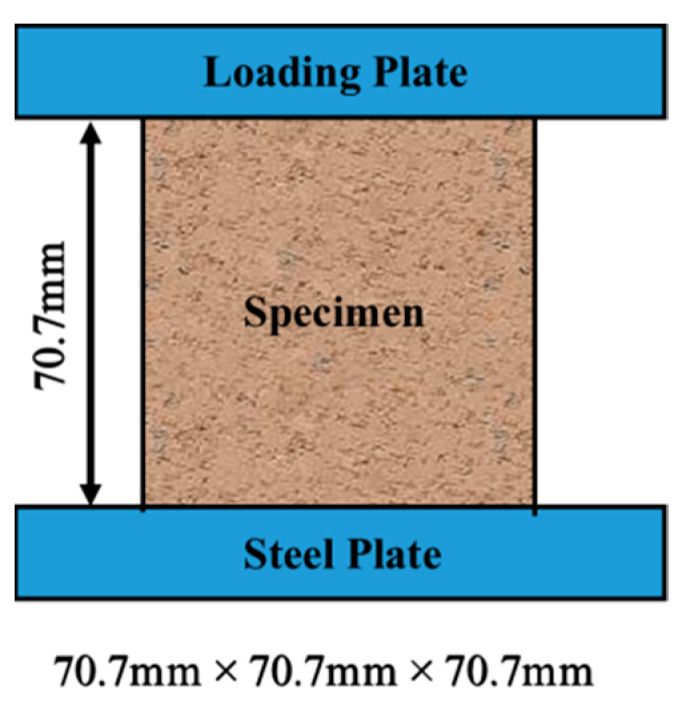
Schematic diagram of compressive strength test.

First, the alkali exciter was prepared by dissolving solid sodium hydroxide in water to obtain a sodium silicate solution, and the solution was left to stand for 24 h. Then, the FA, GGBS, and zeolite powder were mixed and stirred for 1 min. The alkali exciter was added, and the mixture was stirred for an additional 3 min until it was well mixed. After the mixing procedure was done, the fresh geopolymer paste was cast in 70.7 mm × 70.7 mm × 70.7 mm (as shown in Figure 3) cube mold as described in JGJ/T70-2009, and the specimen was obtained after compacting and pounding. Finally, in order to prevent moisture evaporation and effectively maintain the test block, the specimens were placed in a 20 °C constant temperature maintenance box for 24 h and then wrapped in cling film for 3 d and 28 d, respectively, after demolding.

### 4.2. Test Methodology

After the specimens reached the response age, the unconfined compressive strength test was conducted using a WAW-300B microcomputer servo universal testing machine. The average of the test results of three parallel samples was taken as the final result. The instrument used for the scanning electron microscopy (SEM) analysis was a Gemini 500 field emission scanning electron microscope with an acceleration voltage of 3000 V. The instrument used for the X-ray diffraction (XRD) analysis was a D8 Advance X-ray diffraction instrument with a diffraction angle of 2θ, a range of 10–80°, a step size of 0.5 s, and a voltage of 40 kV. The Fourier transform infrared spectroscopy (FTIR) analysis was performed using a Nicolet 5700 FTIR system (KBr press method) in the wavelength range of 4000–400 cm^−1^. The ^29^Si magic angle spinning (MAS) nuclear magnetic resonance (NMR) spectra of the powder samples were obtained at 11.7 T. A Bruker Avance III 400 spectrometer operating at 99.29 MHz and a 5 mm Doty MAS probe with an excitation pulse of 7 μs and a cycle time of 30 s were used. The number of scans ranged from 32 to 87, and the spectra were referenced to tetramethylsilane (TMS).

## 5. Results and Analysis

### 5.1. Experimental Results and Model Analysis

Unconfined compressive strength tests were performed on 17 specimens constructed according to the experimental design conditions. The measured compressive strength of the geopolymer net slurry is presented in Table 3. Among the 17 sets of test data, those with 5 groups were the center points of the design area to estimate the test error.

The tests were conducted using the Design-expert statistical software. The results were obtained after analyzing the relationship between the compressive strength values of the specimens cured for 3 d and 28 d and the three variables A, B, and C (Table 4). In our previous experiments, when the amount of zeolite powder and GGBS is 0 and 20%, the compressive strength at 3 and 28 days is 25.7 MPa and 40.8 MPa. The maximum compressive strength at 3 and 28 days after adding zeolite powder is 40.2 MPa and 50.9 MPa, and the compressive strength is increased by 56.4% and 24.8%. 

In the statistical significance test method, the significance of the difference between the specimens was very significant when the probability *p* of the difference between the samples caused by sampling error was less than 0.01. *p* value between 0.01 and 0.05 was considered significant; *p* > 0.05 was not significant [22]. The *p* value usually measures the misfit error between the model function f and the proper function. In the case of *p* < 0.05, the error was significant, and the fitted model equation was invalid. The larger the *p* value (which does not exceed 1), the better the effect of the fitted model equation [23,24]. As can be seen from Table 4, for the 3 d and 28 d compressive strength models, the quadratic polynomial model had the smallest *p* value (<0.0001). The more significant *p* value of the loss-of-fit test analysis indicated that the model was very significant. The fitted model equation produced the best results, so the quadratic polynomial model was used.

### 5.2. Establishment of the Regression Equation and Merit Search Test

After fitting the test data in Table 3 using the multiple regression method and response surface methodology, the 3 d compressive strength Y_3d_ and 28 d compressive strength Y_28d_ regression models were obtained.

Y_3d_ = 39.16 + 0.27A + 1.26B + 1.59C − 0.88AB + 0.32AC + 0.30BC − 2.73A^2^ − 3.06B^2^ − 7.61C^2^ (1)

Y_28d_ = 49.78 − 1.10A + 0.61B + 1.59C − 0.48AB + 0.27AC − 0.25BC − 4.04A^2^ − 2.02B^2^ − 5.46C^2^ (2)

The F-test is a significance test of the regression model as a whole, and *p* is the probability. When the F value is more extensive and the *p* value is smaller, the more significant the model is, i.e., the smaller the probability that the original hypothesis of the model does not hold, the smaller the error of the simulation [25]. As can be seen from Table 5, the *p* value of the 3 d and 28 d compressive strength models were 0.0004 and <0.0001, respectively, and the F values were 18.84 and 39.24, respectively, which were both highly significant. The 28 d compressive strength was particularly significant. Among the three single factors (A, B, and C) for the compressive strength model, the alkali exciter modulus factor was very significant, but the other two factors were less significant. For example, zeolite powder dosage had a particularly significant effect on the 28 d compressive strength but a less significant effect on the 3 d compressive strength. In contrast, alkali exciter dosage had the opposite trend, with a more significant effect on the 3 d than the 28 d compressive strengths. The overall effects on the compressive strength were ranked as follows: C > B > A.

The correlation coefficient R^2^ between the inferred value of the model and the actual value of the test, the coefficient of variation, and the signal-to-noise ratio were used to measure the credibility of the model. When R^2^ is larger, the coefficient of variation is smaller, and the signal-to-noise ratio is >4, indicating that the test is more reliable. As is shown in Table 4, for the 3 d and 28 d samples, the R^2^ values were 0.9604 and 0.9806, respectively, the corrected R^2^ values were 0.9094 and 0.9556, and the coefficients of variation of the model were 4.05% and 1.95%, respectively. The signal-to-noise ratios were 11.536 and 18.665 (as shown in Table 6), respectively, indicating that the test model had high credibility [26].

**Figure 4:**
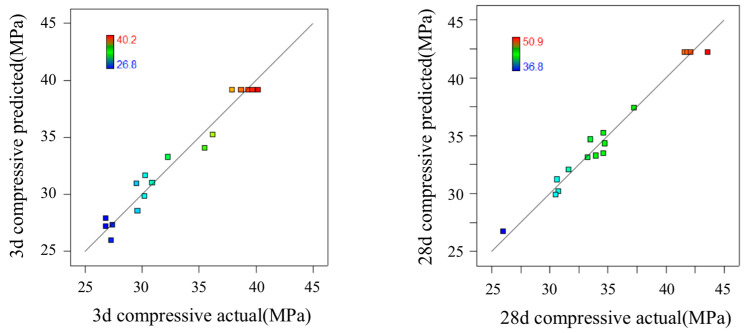
The relationship of predicted and actual values (3/28 d).

**Figure 5:**
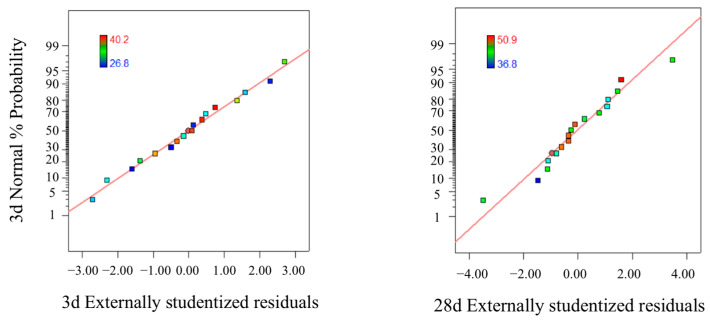
Normal probability plots of residuals (3/28 d).

**Figure 6:**
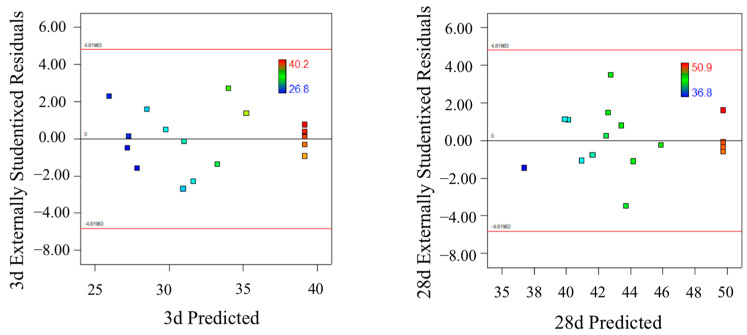
Plot of residuals against predicted response (3/28 d).

Figure 4 displays the relationship between the forecast values and the experimental values (predicted vs. actual) which are distributed comparatively adjacent to the straight line. It is seen that the experimental results are in good agreement with the predicted. Figure 5 is the normal probability distribution diagram of residual, where they lie rationally close on a straight line, which suggests the errors are distributed normally and no digression of the variance. Figure 6 is the pattern of residual and forecast value (residuals vs. predicted). The general trend is that the plot is irregularly distributed, suggesting that the model does not show any violation of independence or that the variance is constant for every response value. All of the above show that the presented regression equation is appropriate to be used for the prediction of 3/28 d compressive strength, and the model is efficiently functional for optimization of the preparation parameters of FA-GGBS geopolymer [27].

### 5.3. Response Surface Interaction Analysis

**Figure 7:**
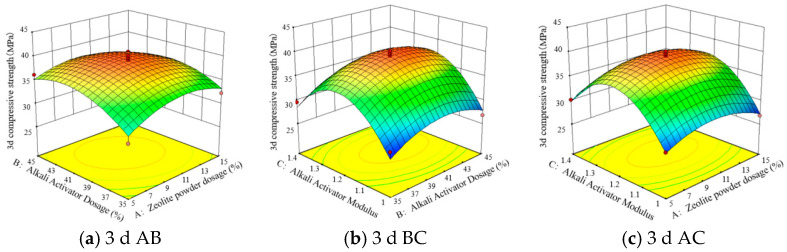

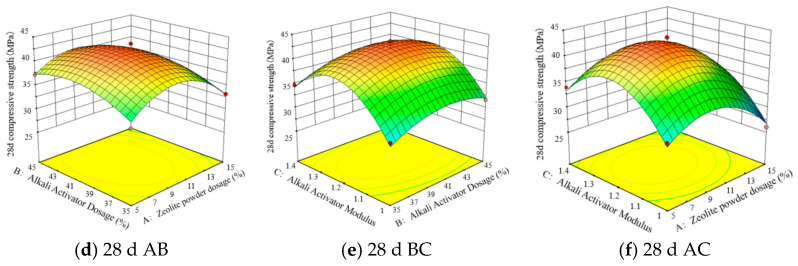
Response surfaces for the experimental model.

**Figure 8:**
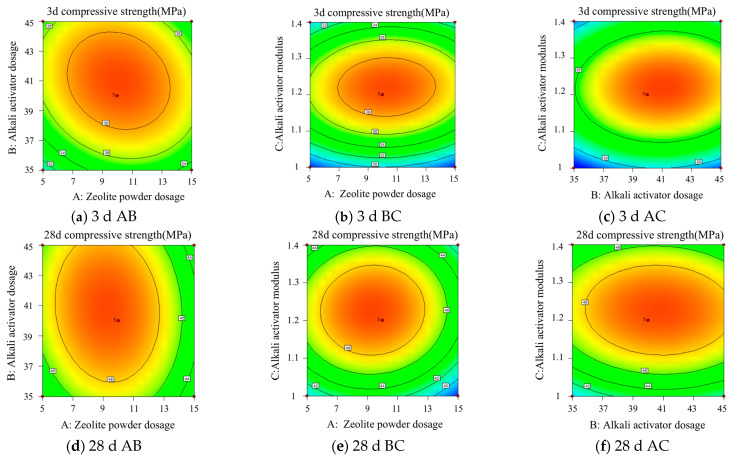
Contour plot for the experimental model.

Figure 7 and Figure 8 show the response surfaces and contour plots of the effects of any two factors from tests A, B, and C on the compressive strength at 3 d and 28 d when interacting with each other. As can be seen from Figure 7a, in the 3 d test, when the modulus of the alkali exciter was 1.2, as the alkali exciter dosage increased, the compressive strength initially increased and then decreased; when the alkali exciter dosage was about 40.3%, the compressive strength reached the peak val-ue. Similarly, as the zeolite powder dosage increased, the compressive strength exhibited the same change trend; when the zeolite powder dosage was about 13.3%, the compres-sive strength reached the peak value. As can be seen from Figure 7b,c, for the 3 d samples, 13.3% zeolite powder, 40.3% alkali exciter, and an alkali exciter modulus of 1.2 were the best values, and the compressive strength was the highest under these conditions. Similarly, as can be seen from Figure 7d–f, for the 28 d samples, the best values were 13.3% zeolite powder, 40.3% alkali exciter, and an alkali exciter modulus of 1.2. Additionally, the 3D response surface in Figure 7 is steep, and the contour in Figure 8 is elliptical. These figures represent a relatively significant interaction between the two factors. 

## 6. Micro-Mechanical Analysis

To further investigate the effect of zeolite powder as an external admixture on the microstructure of the geopolymer specimens, the specimens were re-prepared according to the optimized fitting ratio of the response surface model, and a zeolite powder admixture of 0% was used as the control group for compressive strength and micromechanical analysis. The three specimens were labeled Z0, Z13, and Z20 (Table 7).

### 6.1. Scanning Electron Microscopy Analysis

**Figure 9:**
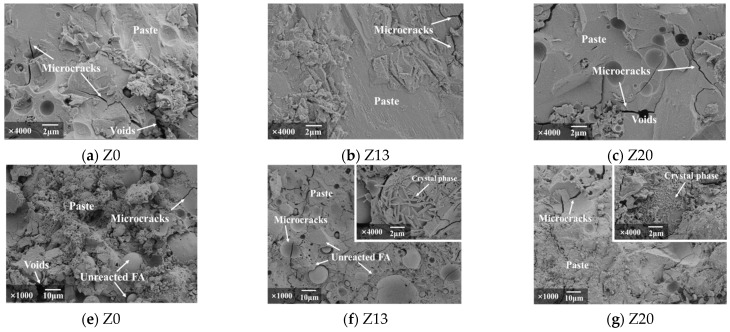
Microstructures of geopolymers with different mixing ratios.

Figure 9 shows the microstructures of the geopolymer specimens under different ratios imaged using an electron microscope. As can be seen from Figure 9a,b, the inter-particle pores are gradually compacted with increasing zeolite powder admixture, which is due to the characteristics of the zeolite powder itself, such as its high specific surface area, porosity, and adsorption capacity, which improve the performance of the FA-GGBS based polymer. Furthermore, this confirms the view of Ozen et al. [17]. The addition of zeolite powder to the geopolymer produced a denser microstructure, lower total pore volume, and optimized pore structure compared to those of ordinary silicate cement. However, when the zeolite powder admixture was increased to 20% (Figure 9c), the pores in the specimens increased again relative to the zeolite powder admixture of 13.3%. This was due to the porosity of the zeolite powder itself. In addition, the excess zeolite powder absorbed more water in the reaction process, leading to a reduction in the amount of water available for the reaction, which was not conducive to the hydration reaction.

The hydroxide ions [28] provided by the alkali exciter eroded and destroyed the vitreous material in the GGBS and FA [29]. With the generation and polymerization of ions such as SiO4^+^ and AlO4^5−^, numerous geopolymer gels (sodium aluminosilicate hydrate, (N-A-S-H) were formed. The gels shown in Figure 9d were mainly flocculent. When the zeolite powder dosage was increased to 13% (Figure 9e), the geopolymer reaction rate increased with increasing zeolite powder dosage. There were abundant needle-like and flaky hydration products attached to the surfaces of the FA particles and closely connected, and the gap between the interface area of the hydration products and FA particles was also tiny. When the dosage amount was increased to 20%, although the needle-like crystalline phase was generated, its distribution was more scattered and not sufficiently closely connected (Figure 9f). Therefore, the measured compressive strength of specimen Z13 was higher than those of specimens Z0 and Z20.

### 6.2. X-ray Diffraction Analysis

**Figure 10:**
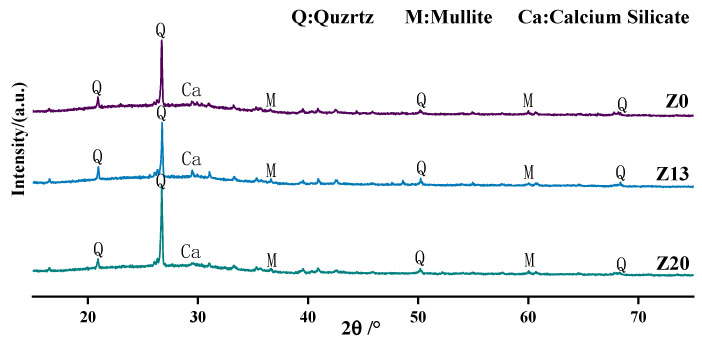
XRD patterns of geopolymers with different mixing ratios.

Figure 10 shows the XRD patterns of the geopolymers with different fitting ratios. The results show that the four groups are composed of crystalline phases such as quartz (SiO_2_), mullite (Al_6_Si_2_O_13_), and dicalcium silicate (Ca_2_SiO_4_). This indicates that the addition of zeolite powder does not affect the crystal composition of the polymer paste. However, the diffraction peak intensities of these minerals in each sample are somewhat different. In addition, as the reaction proceeded, numerous calcium ions were involved in the ground polymerization reaction. Due to the large amount of Ca in GGBS, the content of Ca in the mixture increases, and more C-(A)-S-H is generated under the action of alkaline activator and coexists with N-A-S-H gel. As the zeolite powder dosing increased, the intensities of the diffraction peaks of quartz and mullite weakened which had a better performance. This is reflected in Figure 10 as a broader diffraction peak near a diffraction angle of about 30° [30,31]. As the zeolite powder dosage increased further, the diffraction peak intensity of quartz increased instead, and the diffraction peak intensity of the C-S-H and C-A-S-H gels decreased. It can be concluded that excess zeolite powder is not conducive to the reaction.

### 6.3. Fourier Transform Infrared Spectroscopy

**Figure 11:**
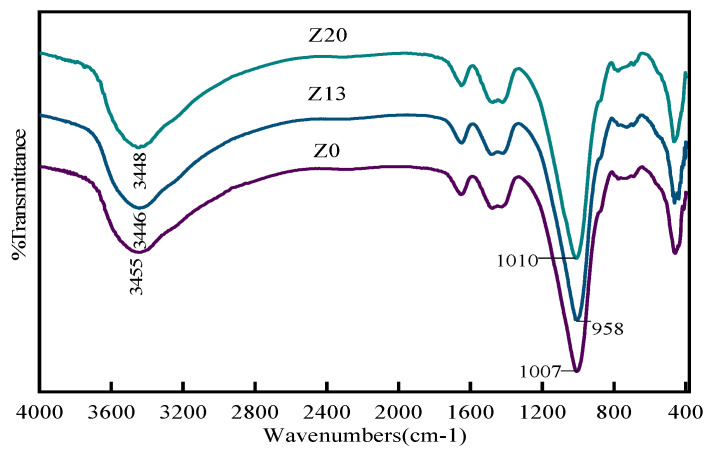
FTIR spectra of geopolymers with different mixing ratios.

Figure 11 shows the FTIR spectra of samples Z0, Z13, and Z20 from bottom to top. According to the principle of infrared analysis, it is known that the stretching vibration frequency of -OH is 3650–3200 cm^−1^, and the peak shape is wider [32]. As can be seen from Figure 5, for the three groups of specimens, the -OH absorption peaks are located at 3455, 3446, and 3448 cm^−1^, the absorption peaks are broad and robust, and the hydroxyl content is greater according to this test analysis for calcium hydroxide and the presence of crystalline water. Among them, Z13 has a smaller absorption peak area than Z0 and Z20, and the absorption peak wave number band is shifted to a lower wave number range. This indicates that the polymerization reaction occurred under optimal zeolite powder dosage, which consumed large amounts of calcium hydroxide and crystalline water. In addition, for all three specimens, Al-O-Si and Si-O-Si asymmetric stretching symmetry peaks appear in the wave number band at around 1000 cm^−1^, corresponding to the products of N-A-S-H and C-A-S-H gels. The Z13 absorption peak wave number band is also shifted to a lower wave number band, indicating that the silica–oxygen bond was replaced by the aluminum–oxygen bond, and more aluminosilicate structures were generated. For the best ratio, the dissolved calcium ions enter the gel during the reaction to replace some of the sodium ions, and due to the combination of calcium ions and silicate monomers, the molar ratio in the gel phase increases, and the products tend to shift to calcium sodium aluminosilicate hydrate (C-N-A-S-H) when the denseness of the products is improved and the strength increases [33].

### 6.4. ^29^Si Nuclear Magnetic Resonance Analysis

**Figure 12:**
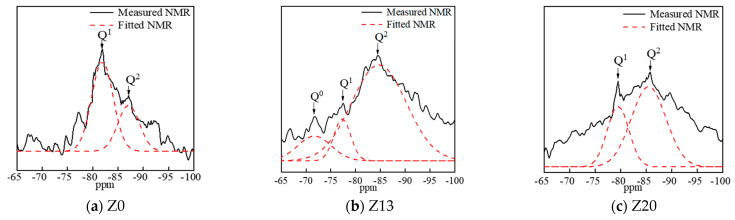
NMR spectra of geopolymers with different ratios.

Figure 12a–c show the ^29^Si NMR spectra of experimental groups Z0, Z13, and Z20. The dashed lines are the fitted curves after performing split-peak fitting. From the structural unit theory of silicates [34], the chemical environment in which ^29^Si is located is represented by Qn, and n values of 0–4 represent the number of oxygen atoms co-joined between one siloxatetrahedron and the surrounding siloxatetrahedra. The chemical shifts of Q^1^, which exists as a short-chain dimer, are −76 to −82 ppm, and the chemical shifts of Q^2^, which exists as a sterically long-chain form, are −82 to −88 ppm. The resonance peaks of Q^1^ and Q^2^ appeared in Z0, Z20, and Z20, and the intensity of the Q^1^ resonance peak decreased in the order Z0 > Z20 > Z13, while the intensity of the Q^2^ resonance peak increased in the order Z13 > Z20 > Z0. The Q^2^ resonance peak is more substantial in the Z13 specimen, and the C-S-H gel generated at this ratio has more C-S-H in long chains with steric linkage [35,36,37]. The gel polymerization degree is more significant, so its intensity is naturally more vigorous.

## 7. Conclusions

In the present study, the effect of using zeolite powder as an external admixture on the performance of FA-GGBS geopolymer has been investigated. A series of experiments were carried out to study the unconfined compressive strength based on the response surface methodology, and the microscopic testings of SEM, XRD, FTIR, and NMR were carried out to reveal the reaction mechanism.

The Box–Behnken test design method of response surface analysis method was used to establish the relationships between the three factors of zeolite powder dosage, alkali exciter dosage, and alkali exciter modulus, and the two response values of 3 d compressive strength and 28 d compressive strength. Based on the experiments, it was found that the highest compressive strengths of the 3 d and 28 d samples were 40.2 MPa and 50.9 MPa. Compared with the control group without zeolite powder, the compressive strength of the 3- and 28-day groups increased by 56.4% and 24.8%. 

Quadratic polynomial equations for 3- and 28-day curing compressive strength were obtained, which described the interaction between independent variables such as zeolite powder dosage, alkali exciter dosage, and alkali exciter modulus. According to the correlation co-efficient and the comparison between the test value and the predicted value, the model al-so presented a satisfactory fit to the factual data. The maximum compressive strengths at 3 d and 28 d were taken as the target optimization values; the optimum values of the zeo-lite powder dosage, alkali exciter dosage, and alkali exciter modulus were determined to be 13.3%, 40.3%, and 1.2, respectively. Additionally, the response surfaces and contour plots of the impact of any two of the three factors A, B, and C on the compressive strength during the interaction were obtained.

Through SEM, FTIR, XRD, and NMR analysis, it was found that the advantages of zeolite powder reduced the total pore volume, optimized the pore structure, made the geopolymer’s microstructure denser, and improved the strength of the geopolymer. However, excessive zeolite powder dosage led to a high-water absorption rate during the reaction process, which was not conducive to the hydration reaction.

Overall, the results of this study indicated that as an external admixture partially replacing FA, zeolite powder could significantly improve the performance (compressive strength) of a FA-GGBS geopolymer, particularly the microstructure of the geopolymer.

## Figures and Tables

**Table 1:** Chemical compositions of FA, blast furnace GGBS, and zeolite powder (wt%).

Chemical Component	Al_2_O_3_	SiO_2_	Fe_2_O_3_	CaO	MgO	TiO_2_	K_2_O	Loss on Ignition
FA	23.67	56.96	4.63	1.50	1.50	2.86	1.73	7.15
GGBS	16.32	36.10	1.28	30.58	9.32	2.94	0.53	2.93
Zeolite	15.47	68.90	0.95	2.32	1.95	0.09	2.16	8.16

**Table 2:** Chemical composition of water glass.

Modulus	Na_2_O (wt%)	SiO_2_ (wt%)	H_2_O (wt%)
3.3	8.3	26.5	65.2

**Table 3:** Design and results of the Box–Behnken test.

Sample	Independent Variable			Compressive Strength (MPa)	
	A	B	C	3 d	28 d
1	5	0.35	1.2	29.5	42.8
2	15	0.35	1.2	32.3	42.6
3	5	0.45	1.2	36.2	45.8
4	15	0.45	1.2	35.5	43.7
5	5	0.40	1	27.4	40.6
6	15	0.40	1	26.8	36.8
7	5	0.40	1.4	30.2	43.2
8	15	0.40	1.4	30.9	40.5
9	10	0.35	1	27.3	40.4
10	10	0.45	1	26.8	41.3
11	10	0.35	1.4	29.6	43.8
12	10	0.45	1.4	30.3	43.7
13	10	0.40	1.2	39.3	49.5
14	10	0.40	1.2	37.9	49.3
15	10	0.40	1.2	38.7	49.5
16	10	0.40	1.2	39.7	50.9
17	10	0.40	1.2	40.2	49.7

**Table 4:** Comprehensive analysis of various compressive strength models.

	Source	Sequential *p*-Value	Lack of Fit *p*-Value	Adjusted R-Squared	Predicted R-Squared	Evaluate
3 d	Linear	0.7531	0.0010	−0.1260	−0.4209	
	2FI	0.9903	0.0005	−0.4481	−1.5958	
	Quadratic	<0.0001	0.0729	0.9194	0.6828	Suggested
	Cubic	0.0729		0.9676		
28 d	Linear	0.6495	0.0006	−0.0895	−0.3220	
	2FI	0.9958	0.0003	−0.4077	−1.3132	
	Quadratic	<0.0001	0.1644	0.9556	0.7772	Suggested
	Cubic	0.1644		0.9756		

**Table 5:** Analysis of variance of simulation equation.

Source	DOF	Mean Square		F Value		*p* Value	
		3d	28d	3d	28d	3d	28d
**Model**	**9**	**42.00**	**29.41**	**18.84**	**39.24**	**0.0004**	**<0.0001**
A	1	0.61	9.68	0.27	12.92	0.6185	0.0088
B	1	12.75	3.00	5.72	4.01	0.0481	0.0855
C	1	20.16	18.30	9.04	24.42	0.0197	0.0017
AB	1	3.06	0.90	1.37	1.20	0.2795	0.3088
AC	1	0.42	0.30	0.19	0.40	0.6764	0.5454
BC	1	0.36	0.25	0.16	0.33	0.6998	0.5816
A2	1	31.38	68.72	14.08	91.71	0.0072	<0.0001
B2	1	39.30	17.10	17.63	22.81	0.0040	0.0020
C2	1	243.52	125.75	109.24	167.81	<0.0001	<0.0001
Residual	7	2.23	0.75				
Lack of Fit	3	4.14	1.20	5.18	2.91	0.0729	0.1644
Pure Error	4	0.80	0.41				

**Table 6:** Model reliability test analysis.

Group	Std. Dev. (MPa)	Mean /MPa	R^2^	Adjusted R^2^	Predicted R^2^	Press	C.V. (%)	Adeq Precision
3 d	1.49	32.86	0.9604	0.9194	0.6828	198.74	4.05	11.536
28 d	0.87	44.36	0.9806	0.9556	0.7772	60.14	1.95	18.665

**Table 7:** Mechanism analysis test group.

Sample	A (%)	B (%)	C
Z0	0	40	1.2
Z13	13	40	1.2
Z20	20	40	1.2

## Data Availability

Details on all data supporting the reported results can be obtained in Table 1, Table 2, Table 3, Table 4, Table 5, Table 6 and Table 7 and Figure 1, Figure 2, Figure 3, Figure 4, Figure 5 and Figure 6 of this original manuscript.
